# Spatial–temporal variation of extreme precipitation in the Yellow–Huai–Hai–Yangtze Basin of China

**DOI:** 10.1038/s41598-023-36470-0

**Published:** 2023-06-08

**Authors:** Lichuan Wang, Jianhua Wang, Fan He, Qingming Wang, Yong Zhao, Peiyi Lu, Ya Huang, Hao Cui, Haodong Deng, Xinran Jia

**Affiliations:** 1 State Key Laboratory of Hydrology-Water Resources and Hydraulic Engineering, Hohai University, Nanjing, 210098 China; 2 State Key Laboratory of Simulation and Regulation of Water Cycle in River Basin, China Institute of Water Resources and Hydropower Research, Beijing, 100038 China; 3 College of Hydrology and Water Resources, Hohai University, Nanjing, 210098 China; 4 College of Oceanography, Hohai University, Nanjing, 210098 China

**Keywords:** Climate sciences, Hydrology

## Abstract

Climate warming leads to frequent extreme precipitation events, which is a prominent manifestation of the variation of the global water cycle. In this study, data from 1842 meteorological stations in the Huang–Huai–Hai–Yangtze River Basin and 7 climate models of CMIP6 were used to obtain the historical and future precipitation data using the Anusplin interpolation, BMA method, and a non-stationary deviation correction technique. The temporal and spatial variations of extreme precipitation in the four basins were analysed from 1960 to 2100. The correlation between extreme precipitation indices and their relationship with geographical factors was also analysed. The result of the study indicates that: (1) in the historical period, CDD and R99pTOT showed an upward trend, with growth rates of 14.14% and 4.78%, respectively. PRCPTOT showed a downward trend, with a decreasing rate of 9.72%. Other indices showed minimal change. (2) Based on SSP1-2.6, the intensity, frequency, and duration of extreme precipitation changed by approximately 5% at SSP3-7.0 and 10% at SSP5-8.5. The sensitivity to climate change was found to be highest in spring and autumn. The drought risk decreased, while the flood risk increased in spring. The drought risk increased in autumn and winter, and the flood risk increased in the alpine climate area of the plateau in summer. (3) Extreme precipitation index is significantly correlated with PRCPTOT in the future period. Different atmospheric circulation factors significantly affected different extreme precipitation indices of FMB. (4) CDD, CWD, R95pD, R99pD, and PRCPTOT are affected by latitude. On the other hand, RX1day and RX5day are affected by longitude. The extreme precipitation index is significantly correlated with geographical factors, and areas above 3000 m above sea level are more sensitive to climate change.

## Introduction

Global warming is accelerating the hydrological cycle, resulting in changes in precipitation, evaporation, tropospheric water vapor, runoff, and other elements. These changes lead to increasingly frequent extreme climate events^1–6^. Extreme precipitation, has a pronounced impact on the natural environment, human safety, and social and economic development^7–10^. Extreme precipitation is characterized by its suddenness and destructiveness, making it prone to inducing or causing disasters^11,12^. The frequency of extreme precipitation events also affects the randomness of the water supply, allocation and dispatching of water resources^13,14^. Moreover, it is expected that by the end of the twenty-first century, global surface temperatures will rise by 1.4–5.8 °C, further increasing the frequency and intensity of extreme precipitation^15^. Therefore, extreme precipitation events have attracted extensive attention due to their negative effects and the disastrous losses associated with them.

Climate change has significant impacts on both global and regional precipitation. However, the response of climate change differs from region to region and specific climatic zones, leading to considerable spatial heterogeneity^16–19^. Moreover, due to variations in geographical conditions, the natural environment, and human activities, extreme precipitation changes also vary across different regions^20–22^. The four basins (FMB) composed of the Yangtze River Basin (YTRB), Yellow River Basin (YLRB), Huaihe River Basin (HURB), and Haihe River Basin (HARB), has an uneven distribution of water resources. The south and east regions of the basin have more water resources, while the north and west regions have less. To address this issue, China has implemented series of inter basin water transfer projects to connect the FMB, making it the largest and most complex area for water supply and consumption in China. These projects include the South-to-North Water Transfer Project and the Yellow River Diversion Project. Thus, changes in extreme precipitation events significantly affect the water resource status of FMB and increase the uncertainty of water supply and usage in the catchment^23^.

Climate change presents a significant challenge to China’s water security, as extreme precipitation events become more frequent and intense^24,25^. For instance, in 2012, heavy precipitation in Beijing, and more recently, in Henan in 2021, led to billions of dollars of economic losses^26,27^. Studies have shown a sharp increase in the frequency of extreme precipitation events since the 1990s, with a clear inter-annual variation trend^28^. As temperatures rise, extreme precipitation is projected to double with each 0.5 °C increase^29^. Researchers have discovered spatio temporal variation patterns in extreme precipitation events in northeast China, southwest China, YTRB, YLRB, and other regions^30^ highlighting the obvious regional characteristics of extreme precipitation in China^1,31,32^. Although individual studies on watersheds, climate regions, and the FMB's individual basins have been conducted, there is a need for a single study that combines the individual basins of the FMB to reveal spatial variation patterns in extreme precipitation. Global climate models (GCMs) are credible tools for analysing future extreme precipitation and can effectively incorporate extreme disasters. Currently, GCM data sets under phase 6 (CMIP6) of the coupled model mutual comparison project can be analysed to infer potential future extreme precipitation changes under various Shared Socioeconomic Pathways (SSPs)^33^. Several factors affect the spatial and temporal characteristics of regional precipitation, and previous discussions have focused on the impact of the atmospheric circulation model on the region. The terrain of the FMB is complex and diverse, with a multi cascade distribution with a total variation in elevation of approximately 5400 m. The impact of geographical factors on extreme precipitation in the FMB is significant. Therefore, it is necessary to analyse past and projected future extreme precipitation in the FMB and study the impacts of geographical factors and atmospheric circulation on these events.

The primary aims of this study were to achieve a more comprehensive understanding of the following: (1) the factors that influence and contribute to extreme precipitation events in the FMB; (2) the spatial distribution patterns of extreme precipitation under various Shared Socioeconomic Pathway (SSP) scenarios; (3) the correlation between extreme precipitation indices; and (4) the factors that impact extreme precipitation events based on the correlation between extreme precipitation, geographical conditions, and atmospheric circulation.

## Data and methods

### Study area

**Figure 1:**
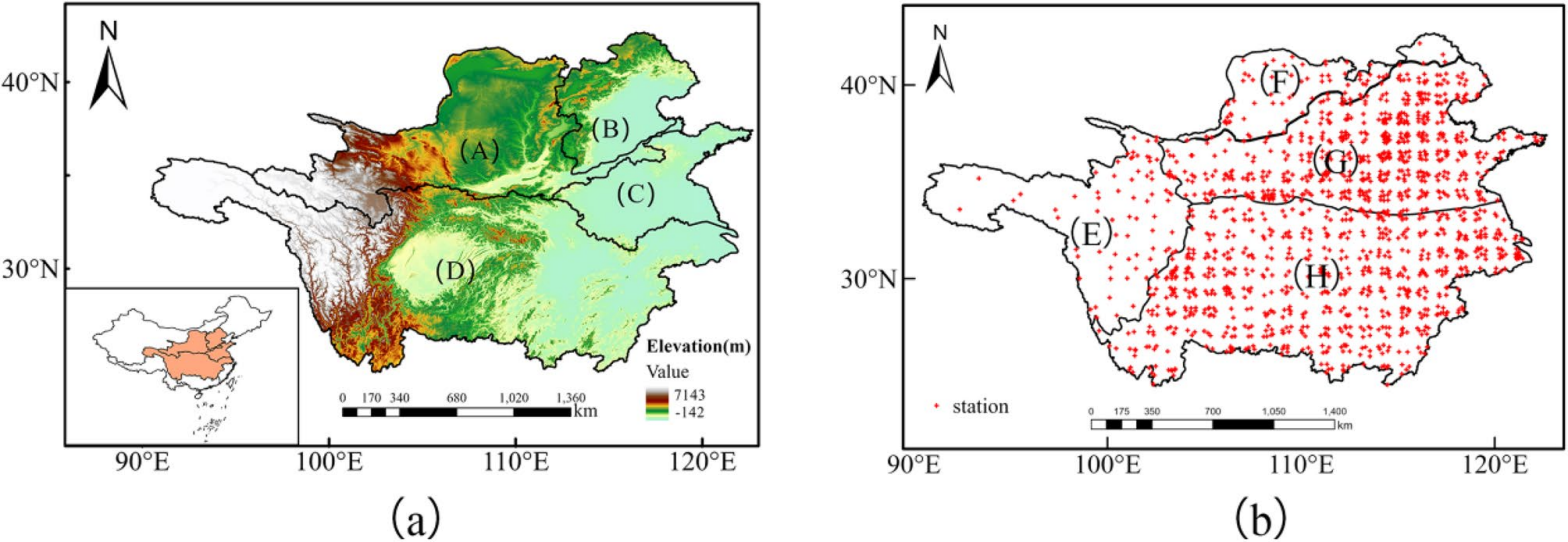
(**a**) Study area and (**b**) location map of Meteorological Station. (**A**) Yellow River basin, (**B**) Haihe River basin, (**C**) Huaihe River basin, and (**D**) Yangtze River basin, (**E**) Plateau alpine climate region, (**F**) Temperate continental climate region, (**G**) Temperate monsoon climate region, (**H**) Subtropical monsoon climate region. Maps were generated using Qgis 3.30 (https://qgis.org/en/site/).

As shown in Fig. 1, the research area selected for this study is in China and includes four major rivers: the Yellow River (A), Haihe River (B), Huaihe River (C), and Yangtze River (D). The boundaries of the area are approximately 24°27′–43°00′ N and 90°13′–122°60′ E, covering an area of 3,233,000 km^2^.

The study area includes plains, hills, and mountains. The western part of the study area has a plateau mountain climate (E); while the northern part has a temperate continental climate (F); the central part experiences a temperate monsoon climate (G); and the southern part has a subtropical monsoon climate (H). The eastern coastal region is a transition region with a mix of climates, including a temperate zone continental climate transitioning to a temperate monsoon climate, a plateau mountain climate transitioning to a subtropical monsoon climate, and a plateau mountain climate changing to a sub-tropical monsoon climate zone. The plains of the Tibetan plateau transition to the terrain, ocean, and inland areas of east China.

Water scarcity is a significant issue in the Huang–Huai–Hai basin, which provides the country with 7% of its water supply, despite being home to 34% of its population, and contributing 38% of its GDP. The YTRB region, which is dominated by plains, has an average annual precipitation of approximately 800–1943 mm. The region is known for its fertile soil and abundant lakes and water systems.

### Data

For the historical scenario, we used daily precipitation data from 1960 to 2014 obtained from 2416 meteorological stations in China. The data were obtained from the National Meteorological Science Data Centre (https://data.cma.cn/) and included data from 1842 sites in our research area. We excluded missing years to ensure data integrity and consistency.

To obtain future predicted precipitation data under various climate scenarios, we used output data from GCMs available in the CMIP6 (https://esgf-node.llnl.gov/projects/cmip6/) We considered seven climate patterns in this study (Table 1), including scenarios SSP1-2.6, SSP3-7.0, and SSP5-8.5. Scenario SSP1-2.6 assumes a sustainable development scenario with global warming of less than 1.5 °C, covering the period 2015–2100. Scenario SSP3-7.0 is a moderate scenario with approximately 60–80 billion tons of CO_2_ emissions and 4.5 °C of warming by the end of the twenty-first century. Scenario SSP5-8.5 represents the worst-case scenario, assumes the world adopts no policies to deal with climate change, and allowing for free development of global warming. Under this scenario, global annual CO_2_ emissions are expected to exceed 120 billion tons, and the average temperature rise could reach 5–6 °C by the end of the twenty-first century.

**Table 1:** CMIP6 mode used in this study.

Model name	Country	Units	Resolutions
			Lat. × Long
BCC-CSM2-MR	China	BCC	1.125° × 1.1°
CanESM5	Canada	CCCMA	2.8125° × 2.8°
CNRM-CM6-1	France	CNRM	1.4° × 1.4°
INM-CM4-8	Russia	INM	2° × 1.5°
IPSL-CM6A-LR	France	IPSL	2.5° × 1.3°
MRI-ESM2-0	Japan	MRI	1.125° × 1.1°
UKESM1-0-LL	UK	UKESM	1.875° × 1.25°

Given the impact of global climate change and regional atmospheric circulation background, we included 12 atmospheric circulation indicators in our analysis to investigate their relationship with atmospheric circulation and FMB extreme precipitation events. To do so, we obtained six large-scale atmospheric circulation patterns from the Earth System Research Laboratory of the Physical Sciences Division of the United States National Oceanic and atmospheric administration (https://www.esrl.noaa.gov/psd/data/climateindices/list/).

These patterns include Atlantic Multidecadal Oscillation (AMO), Arctic Oscillation (AO), North Atlantic Oscillation (NAO), Pacific Decadal Oscillation (PDO), and El Niño Southern Oscillation (ENSO) (including the multivariate ENSO index (MEI) and Nino3.4 sea surface temperature. In addition, we downloaded the East Asian summer monsoon Index (EASMI) and South China Sea summer monsoon index (SCSSMI) from http://lijianping.cn/dct/page/1.

### Data calculations

To begin, both the historical and future data were interpolated into a 25 km × 25 km spatial grid using the Anusplin interpolation method as described in previous studies^34,35^. As climate models have great uncertainty in both emission scenarios and model uncertainty. To reduce the uncertainty, we calculated the average weight of the multi-mode set using the Bayesian model averaging method (BMA)^36^. Furthermore, we corrected any deviations using the non-stationary cumulative distribution function matching technique (CNCDFm)^37^.

For future data, we first interpolated the historical data from seven models into 25 km × 25 km grid, and generated historical data for the new BMA model using the BMA model. We then used CNCDFm to correct the deviations in each model. After conducting 30 cross validation, we concluded that the BMA model was more accurate, smaller, and more reasonable than the observed data. This method was then used to scale down data in the future periods.

The variation correction formula can be expressed using the by Eqs. (1–4):

1 $$ x_{m - p.adjust} = \left( {\begin{array}{*{20}l} {I(x),I(x) > 0} \hfill \\ {g(x),I(x) < 0} \hfill \\ {0,x_{m - p} = 0} \hfill \\ \end{array} } \right. $$

2 $$ I(x) = x_{m - p} + F_{o - c}^{ - 1} \left( {F_{m - p} \left( {x_{m - p} } \right)} \right) - F_{m - c}^{ - 1} \left( {F_{m - p} \left( {x_{m - p} } \right)} \right) $$

3 $$ g(x) = x_{m - p} \times \frac{{F_{o - c}^{ - 1} \left( {F_{m - p} \left( {x_{m - p} } \right)} \right)}}{{F_{m - c}^{ - 1} \left( {F_{m - p} \left( {x_{m - p} } \right)} \right)}} $$

where *x* is observed precipitation variables (*o*) or model (*m*) for a historic training period or future projection period (*p*); *I*(*x*) and *g*(*x*) are EDCDFm and equi-ratio CDFm methods, respectively; *F*(·) is the CDF for a variable, and *F*^-1^(·) is its inverse.

4 $$ f(x;k,\theta ) = x^{k - 1} \frac{{e^{ - x/\theta } }}{{\theta^{k} \Gamma (k)}}\,\,\,\,for\,\,\,\,x > 0\quad and\,\,\,\,k,\,\,\theta > 0 $$

where ${\Gamma }$ (·) is the gamma function; the shape parameter (*k*) and the scale parameter ($\theta$) were estimated by the method of maximum likelihood estimation.

According to research, the improved non-stationary deviation correction technology can eliminate 80% of model deviation and 60% of uncertainty. It can also correct the unreasonable extreme value of the region^37^. In this study, the analysis of extreme precipitation based on this method is more reliable and reasonable than other methods tested.

### Extreme climate index definition

The expert team on climate change detection and indices (ETCCDI; http://cccma.seos.uvic.ca/ETCCDI) has developed a climate change detection and index including 40 indices related to extreme weather. These indices are widely used in the analysis and research of extreme weather events due to their ability to identify weak extreme events, have salience, and exhibit low noise characteristics. In this study, 11 indicators related to extreme precipitation were selected and divided into 3 categories (Table 2). It is widely used to assess climate change due to extreme precipitation events in different parts of the world.

**Table 2:** Extreme precipitation index used in this study.

Type	ID	Name	Definition	Unit
Duration indices	CDD	Consecutive dry days	Maximum number of consecutive days with RR (daily precipitation) < 1 mm	Day
	CWD	Consecutive wet days	Maximum number of consecutive days with RR ≥ 1 mm	Day
Strength index	PRCPTOT	Annual total precipitation	Annual total precipitation in wet days (defined as RR ≥ 1 mm)	mm
	R95pTOT	Extreme precipitation	Annual total precipitation when daily precipitation > 95th percentile in wet days	mm
	R99pTOT	Extreme heavy precipitation	Annual total precipitation when daily precipitation > 99th percentile in wet days	mm
	Rx1day	Max 1-day precipitation	Maximum total 1-day precipitation	mm
	Rx5day	Max 5-day precipitation	Maximum total 5-days precipitation	mm
Frequency index	R10mm	Number of heavy precipitation days	Number of days with daily precipitation ≥ 10 mm	Day
	R20mm	Number of very heavy precipitation days	Number of days with daily precipitation ≥ 20 mm	Day
	R95pD	Annual precipitation fraction due to very wet days	Number of days when daily precipitation > 95th percentile of precipitation on wet days	Day
	R99pD	Annual precipitation fraction due to extremely wet days	Number of days when daily precipitation > 99th percentile of precipitation on wet days	Day

### Linear trend and significance tests

Sen’s nonparametric slope estimation method^38^ was used to calculate the variation trend of meteorological features. The formula is:

5 $$ Q_{i} = \frac{{x_{j} - x_{k} }}{j - k}\quad i = 1, \ldots N $$

where *Q*_*i*_ reflects the steepness of the trend in the data; *x*_*j*_ and *x*_*k*_ are the time series values of j and k samples respectively (*j* > *k*), $N = \frac{{n\left( {n - 1} \right)}}{2}$.

The values were arranged from smallest to largest, then the slope of the median Sen’s was estimated as:

6 $$ Q_{med} = \left\{ {\begin{array}{*{20}l} {Q_{{\left[ {\left( {N + 1} \right)/2} \right]{\kern 1pt} }} } \hfill & {N\,{\text{is}}\,\,{\text{odd}}} \hfill \\ {\frac{{Q_{{\left[ {N/2} \right]}} + Q_{{\left[ {\left( {N + 2} \right)/2} \right]}} }}{2}} \hfill & {N\,{\text{is}}\,\,{\text{even}}} \hfill \\ \end{array} } \right. $$

where *Q*_*med*_ reflects the steepness of the trend in the data, *Q*_*me*d_ is greater than zero, the sample has an upward trend; otherwise, it has a downward trend.

In order to eliminate the influence of time series autocorrelation on the results, Modified Mann–Kendall test (MMK test) was used to calculate the significance level of the trend^39^. The formula is:

7 $$ S = \mathop \sum \limits_{i = 1}^{n - 1} \mathop \sum \limits_{j = i + 1}^{n} sgn\left( {X_{j} - X_{i} } \right) $$

where *X*_*j*_ and *X*_*i*_ is *j* and *i* the time series value of the sample; n is the length of the data set; S is the test statistic.

8 $$ sgn\left( \theta \right) = \left\{ {\begin{array}{*{20}l} { 1} \hfill & {if\,\, \theta > } \hfill \\ 0 \hfill & {if\,\,\theta = 0} \hfill \\ { - 1} \hfill & {if\,\, \theta < 0} \hfill \\ \end{array} } \right. $$

The statistic S is approximately normally distributed when n ≥ 8, with the mean and the variance as follows:

9 $$ E\left[ S \right] = 0 $$

10 $$ V\left( S \right) = \frac{{n\left( {n - 1} \right)\left( {2n + 5} \right) - \mathop \sum \nolimits_{i = 1}^{n} t_{i} i\left( {i - 1} \right)\left( {2i + 5} \right)}}{18} $$

where $t_{i}$ is the number of ties of extent *i*.

11 $$ \left\{ {\begin{array}{*{20}l} {Z = \left( {S - 1} \right)\sqrt {Var\left( S \right)} } \hfill & {S > 0} \hfill \\ {Z = 0} \hfill & {S = 0} \hfill \\ {Z = \left( {S + 1} \right)\sqrt {Var\left( S \right)} } \hfill & {S > 0} \hfill \\ \end{array} } \right. $$

12 $$ p = 0.5 - \emptyset \left( {\left| Z \right|} \right) $$

$$ \left( {\emptyset \left( {\left| Z \right|} \right) = \frac{1}{{\sqrt {2\pi } }}\mathop \smallint \limits_{0}^{\left| Z \right|} e^{{ - \frac{{t^{2} }}{2}}} dt} \right) $$

The MMK trend significance test^40,41^ is represented by statistical parameter Z. When the |Z|≥ 1.96 or higher at the 95% confidence level (*P* < 0.05), the evaluated trend is defined as being statistically significant. Similarly, when |Z|≥ 2.58 or higher at the 99% confidence level (*P* < 0.01), the assessment trend is statistically significant.

### Correlation analysis

The linear relationship between the two variables is used to describe the degree of correlation, and Pearson correlation analysis is used as expressed by Eq. (13):

13 $$ \rho_{X,Y} = corr(X,Y) = \frac{{{\text{cov}} (X,Y)}}{{\sigma_{X} \sigma_{Y} }} = \frac{E[(X - \mu x)(Y - \mu y)]}{{\sigma_{X} \sigma_{Y} }} $$

where *ρ*_*X,Y*_ is correlation; cov(*X,Y*) is Covariance of *X* and *Y*;σ_X_ and σ_Y_ are Standard deviation of *X* and *Y*;*E*(·) is expected value; *X* and* Y* is time series.

Positive correlation means that the correlation value is greater than 0, and negative correlation means that the correlation value is less than 0. The closer the absolute value of correlation to 1, the stronger the correlation.

## Results

### Analysis of time variation trend of extreme precipitation

**Figure 2:**
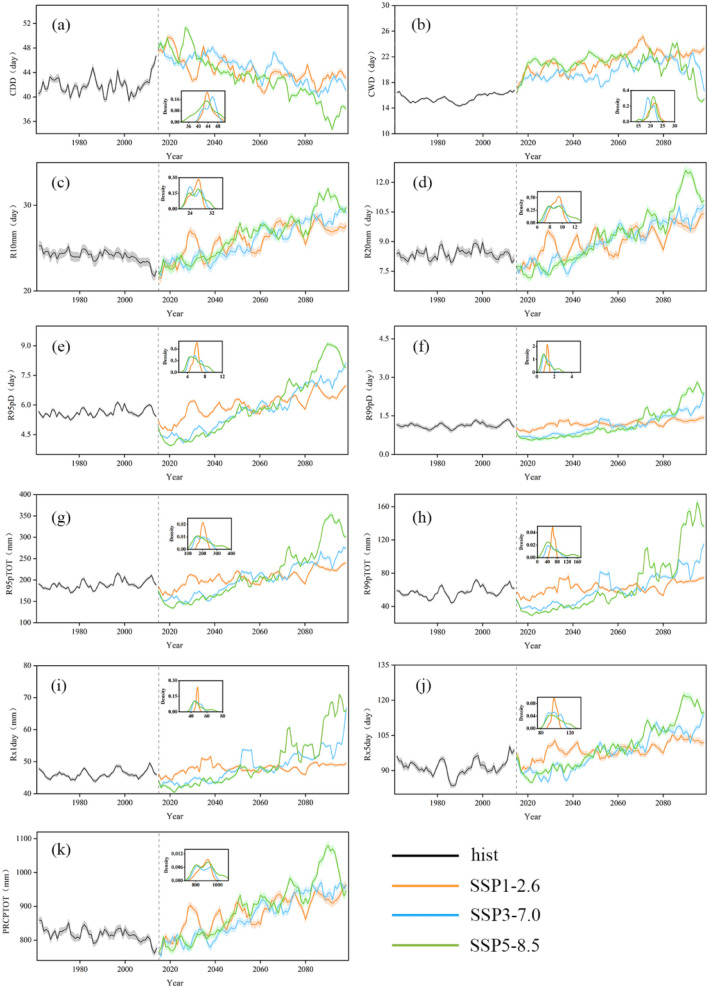
Five-year sliding average values of CDD (**a**), CWD (**b**), R10mm (**c**), R20mm (**d**), R95pD (**e**), R99pD (**f**), R95pTOT (**g**), R99pTOT (**h**), Rx1day (**i**), Rx5day (**j**) and PRCPTOT (**k**) during 1960–2100 across FMB. Solid black curve modelled historical extreme precipitation indices. Orange/blue/green curves denote modelled extreme precipitation indices under SSP1-2.6/SSP3-7.0/SSP5-8.5 scenarios. Shaded areas show 5%-95 confidence interval. The inset figure shows density of each extreme precipitation index.

Figure 2 depicts the trend of the 5-years moving average of eleven extreme indices: CDD (a), CWD (b), R10mm (c), R20mm (d), R95pD (e), R99pD (f), R95pTOT (g), R99pTOT (h), Rx1day (i), Rx5day (j) and PRCPTOT (k) in the FMB during 1960–2100. The results show that the ensemble mean values of PRCPTOT, CWD, R10mm, R20mm, R95pD, R99pD, R95pTOT, R99pTOT, Rx1day and Rx5day all demonstrated a pronounced upward trend. CDD was an exception and showed a continuous and substantial downward trend.

In the historical period, the eleven indices changed slightly: CDD and R99pTOT showed an upward trend with a growth rate of 14.14% and 4.78%, respectively; PRCPTOT showed a downward trend with a rate of 9.72%; while the other indices had minimal change. This shows that in the historical period, the number of dry days and precipitation intensity increased, and the extreme heavy precipitation events were more concentrated.

The study found that the trend of each scenario in the future period, the trend of each scenario is statistically significant, with scenario SSP1-2.6 is the smallest, followed by SSP3-7.0 and SSP5-8.5 is the largest. However, by the mid-twenty-first century, the SSP1-2.6 index is the largest for most models except for CDD and CWD. After the middle of the twenty-first century, SSP5-8.5 has the largest values of all indices, follows by SSP3-7.0 and SSP1-2.6. In general, the indices demonstrate a pronounced increase in extreme precipitation, precipitation intensity, and the number of precipitation days, with a substantial decrease in the number of consecutive dry days. However, CDD is the only index showing a continuous and substantial downward trend, indicating an increase in the number of consecutive dry days. SSP1-2.6 have a development trend comparable to the historical period, with a decreasing CDD rate of 9.20%. The growth rates of other indices are approximately 25%. R95pTOT have the highest growth rate at 30.07%. Under the scenario of SSP3-7.0, the decline rates of CDD and CWD are 14.39% and 8.16% respectively, while the growth rates of other indexes are about 30%. PRCPTOT have the highest growth rate at 31.69%. In the SSP5-8.5 scenario, the reduction rates of CDD and CWD are 20.82% and 16.83% respectively, the rates of increase for R99pTOT, R95pTOT, and PRCPTOT are 372.89%, 72.34%, and 59.90%, respectively. The growth rates of other indices are all higher than 35%. Comparable to the SSP1-2.6 scenario, the CDD index in SSP3-7.0 decreased by 5.19%, while the other indices increased by 5%. SSP5-8.5 have a statistically significant range in variation, and the change rates for all indices were higher than 10%. By the end of the twenty-first century, the CDD index for SSP5-8.5 is 84% of SSP1-2.6, and that for SSP3-7.0 is 96% of SSP1-2.6. The CWD index for SSP5-8.5 is 66% of SSP1-2.6, and that for SSP3-7.0 is 72% of SSP1-2.6. The R99pTOT and Rx1day index for SSP5-8.5 is 2.05 times that of SSP1-2.6, and that for SSP3-7.0 is 1.3 times that of SSP1-2.6. The other indices for SSP5-8.5 are approximately 1.1 times that of SSP1-2.6, while the indices for SSP3-7.0 are approximately 1.03 times those of SSP1-2.6. Therefore, these indices all indicate that, in the future, climate warming will lead to a pronounced increase in extreme precipitation, the number of precipitation days, precipitation intensity, and a substantial decrease in the number of consecutive dry days.

In addition, we analysed the change trend and density distribution of extreme precipitation events in the future period. The results show that the trend of CDD in SSP1-2.6 is -0.05, SSP3-7.0 is -0.07, and SSP5-8.5 is 0.14. The peak density of SSP1-2.6 and SSP5-8.5 is 43.8 d. The SSP3-7.0 peak shift to the right by 46.1 d. The peak value of CWD of SSP1-2.6 and SSP5-8.5 is greater than that of SSP3-7.0 for about 5 days. Moreover, the change trend of PRCPTOT in the SSP1-2.6 scenario is 1.77, is 2.60 in SSP3-7.0, is 3.29 in SSP5-8.5, and other indices show similar trends. The peak values of R10mm, R20mm, R95pTOT, R99pTOT, R95pD, R99pD, Rx1day and Rx5day are high in SSP1-2.6 and low in SSP5-8.5. In terms of density distribution, the tail of each index shows a larger value of SSP5-8.5. These findings suggest that climate change will have varying impacts on extreme precipitation events, depending on the scenario. As a result, it is important to consider these potential impacts when developing policies and strategies to mitigate and adapt to the effects of climate change (Wang et al. 2020).

### Analysis of spatial variation trend of extreme precipitation

**Figure 3:**
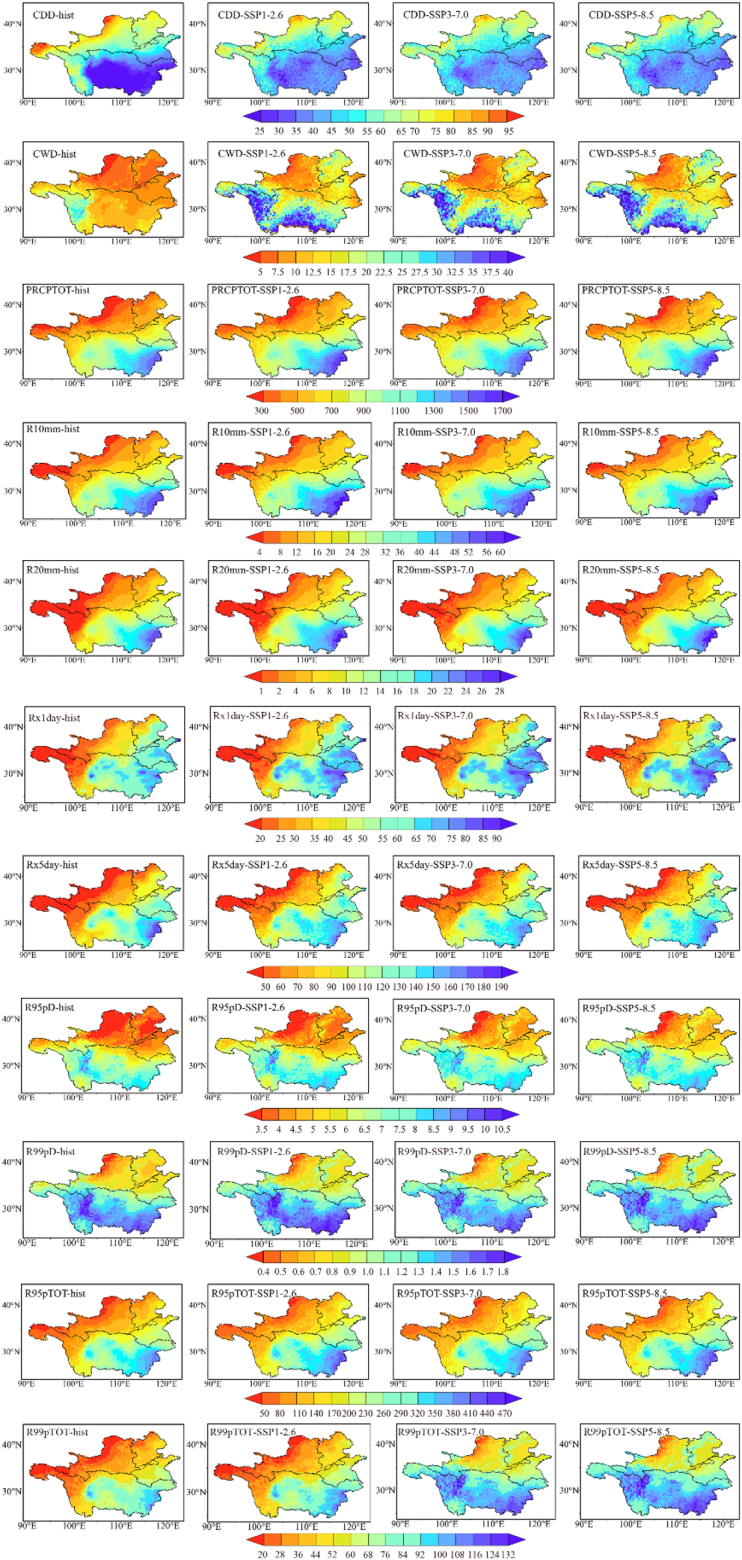
The spatial pattern of average annual extreme precipitation index of the FMB, the first column is hist scenario, the second column is SSP1-2.6, the third column is SSP3-7.0, and the fourth column is SSP5-8.5. Maps were generated using Qgis 3.30 (https://qgis.org/en/site/).

Figure 3 presents the seven extreme precipitation indices (CDD, CWD, PRCPTOT, R10mm, R20mm, Rx1day, Rx5day, R95pD, R99pD, R95pTOT and R99pTOT) for both the historical data and future periods under SSP1-2.6, SSP3-7.0, SSP5-8.5, along with the spatial distribution of the four situations. The spatial distribution characteristics of all extreme precipitation indices in the historical and future periods are similar, showing that the northern portion of the study area was arid with less extreme precipitation, while the southern part was humid with more extreme precipitation events. PRCPTOT, R10mm, R20mm, Rx1day, R95pD, R99pD, R95pTOT and R99pTOT showed a gradual increase in values from northwest to southeast within the FMB, while CDD demonstrated the opposite trend. The value of CWD in the YTRB was larger than that in the Huang–Huai–Hai Basin. Compared with the historical period, the spatial pattern of the future period changed substantially. Continuous wet days increased sharply in the FMB, with the YERB, HURB, and HARB showing a spatial pattern different from that of precipitation. The PRCPTOT, R10mm, R20mm, Rx1day, Rx5day, R95pTOT and R99pTOT showed a trend of substantial growth in the future period, although their changes in value were not significant during the historical period.

The extreme precipitation characteristics in different climate regions were analysed in further detail, considering that the FMB has distinct climate zones. In the altitude is higher than 3000 m in the plateau alpine climate region, the extreme precipitation index changed significantly. The trend with the steepest slope was observed for R95pTOT had (0.12 mm/yr), followed by PRCPTOT (0.05 mm/yr), while CDD and CWD demonstrated negative trends (− 0.04 and − 0.02 mm/yr, respectively). However, in the future period, except for CWD all other indices showed a significant increase with the rise of carbon emissions and worsening of climate warming. The slopes of CDD and CWD showed a decreasing trend, while other Indices increased. PRCPTOT had the steepest slope and showed a significant increase of 1.47, 2.80, and 3.20 mm/yr in the SSP1-2.6, SSP3-7.0, SSP5-8.5 scenarios. The slopes of R95pTOT were 0.58, 1.6, and 2.13 mm/yr, respectively, for the same scenarios.

In the temperate continental and temperate monsoon climate zones, the trends were similar.Except for CDD and CWD, the change trend of these two regions was significant. The change of CDD in the two regions was not significant in the historical period. In the scenario of SSP1-2.6, the temperate continental climate region CWD does not change significantly. Only the trend for CDD was negative. PRCPTOT and R95pTOT showed the steepest slopes. In the subtropical monsoon climate zone, In the future, the change of CWD is not significant, the change of CDD in SSP1-2.6 is not significant. The change of other indexes was significant. SSP3-7.0 showed different regularities in CDD, CWD, and PRCPTOT, with flatter slopes than in the other models.

**Figure 4:**
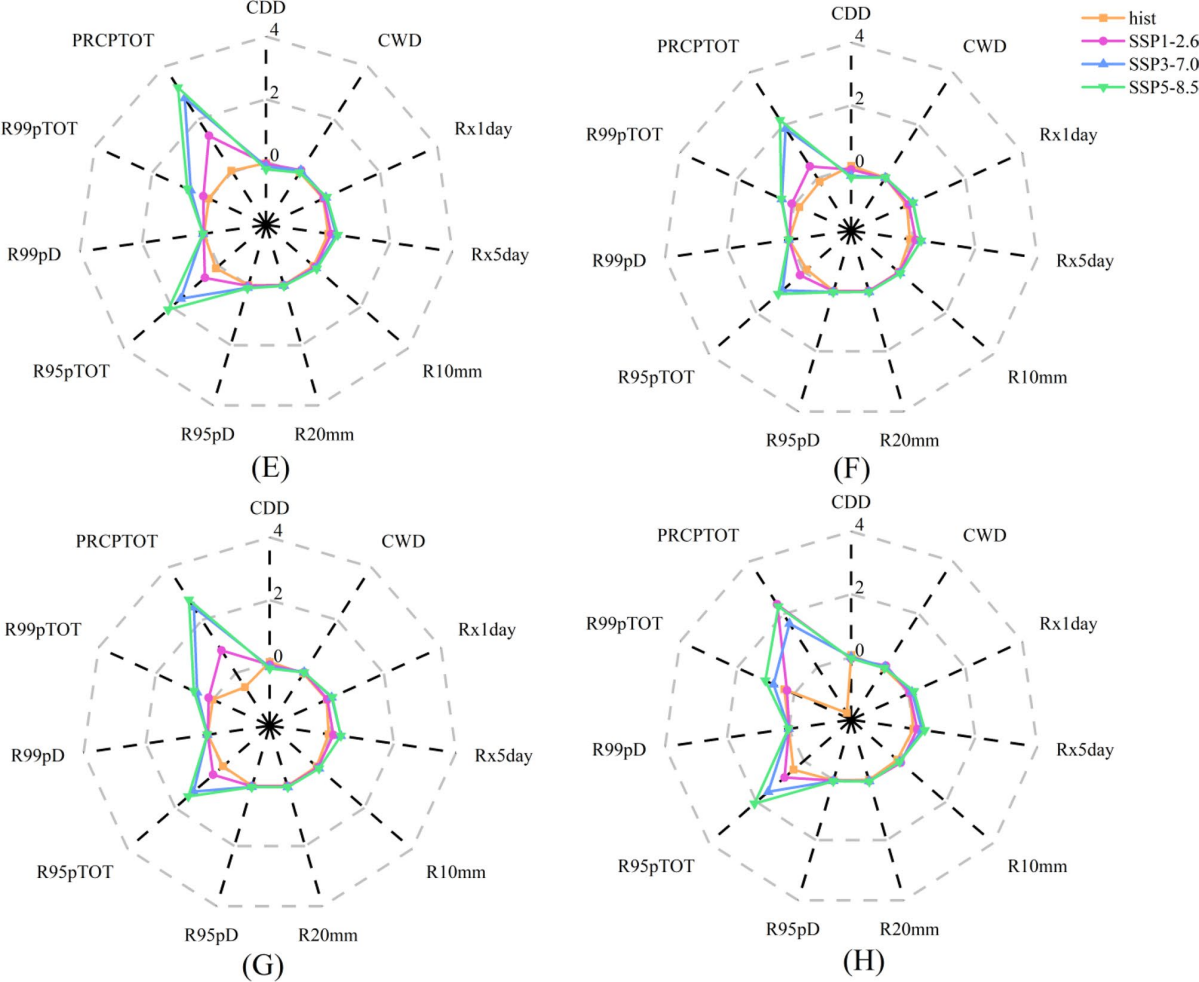
Extreme precipitation index Sen’s slope (**A**) Plateau alpine climate region (**F**) Temperate continental climate region (**G**) Temperate monsoon climate region (**H**) Subtropical monsoon climate region.

Figure 4 illustrates the Sen’s slope values for different extreme precipitation index in various climatic zones. Among the indices, CWD, R10mm, R95pD, and R99pD exhibited relatively stable, with slopes less than 0.1 under various scenarios. However, the CDD index was found to be more sensitive to climate warming in the plateau alpine and temperate continental climates, with a slope reduction of SSP5-8.5 by -0.17 d/yr and -0.25 d/yr, respectively. The slope of Rx1day was lower in SSP1-2.6 than in SSP5-8.5 and was approximately 0.1 d/yr in SSP3-7.0, and approximately 0.2 d/yr in SSP5-8.5. The trend slopes for the temperate continental and temperate monsoon climates were steeper than for the other climate zones. The slope of Rx5day was about twice that of Rx1day, and the rate of change was between 0.1 d/yr and 0.2 d/yr under various scenarios. In the plateau alpine and subtropical monsoon climates, the slopes of R95pTOT and R99pTOT were steeper than those for other climatic regions. Among all the indices, the PRCPTOT was the largest and showed varying trends in the subtropical monsoon climate. The largest slope value was found in SSP1-2.6 (2.37 mm/yr), while the lowest in SSP3-7.0 (1.64 mm/yr), indicating a change of 0.73 mm/yr. The slope value of other climate regions followed an increasing order: SSP1-2.6 < SSP3-7.0 < SSP5-8.5, while decreasing in the order: plateau alpine climate region > temperate monsoon climate zone > temperate continental climate zone. These results imply that in the semi-arid region, specifically the plateau alpine climate with high altitude, is the most sensitive to extreme precipitation, while climate change had minimal impact on the temperate continental climate of the plains. The subtropical monsoon climate in the humid region shows a sensitivity to extreme precipitation resulting from climate change different from that of the semi-arid region.

### Analysis of seasonal variation of extreme precipitation index

Climate change has the potential to significantly impact the precipitation process and alter the structure of precipitation. Extreme precipitation in different climate regions exhibits varying responses to climate change not only in the interannual period but also in distinct seasons, which can have significant implications for the region's water resources. Therefore, to analyse the impact of climate change on extreme precipitation, we focused on three key indices with a high impact on extreme precipitation: PRCPTOT, CDD, and Rx5day. PRCPTOT is a measure of the total wet day precipitation, which is a key factor in assessing the total precipitation. The CDD, on the other hand, is an important index to assess drought conditions. Finally, Rx5day is an important index to reflect flood risks.

**Figure 5:**
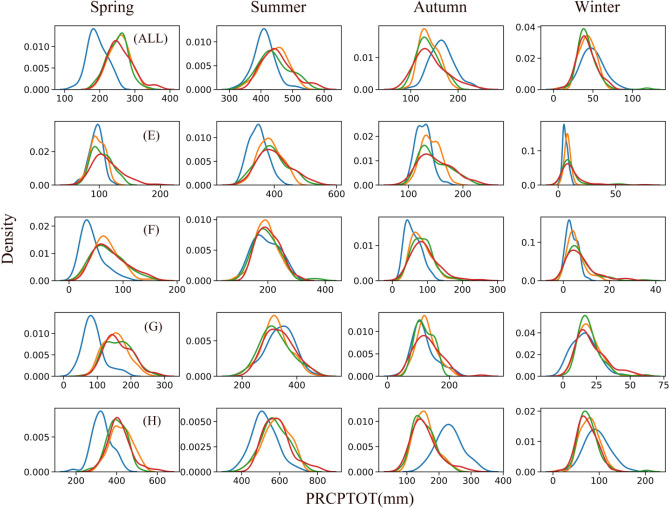
Probability density map of PRCPTOT in different seasons (Blue is hist scenario, orange is SSP1-2.6 scenario, green is SSP3-7.0, and red is SSP5-8.5 scenario) (ALL) F MB; (**E**) Plateau alpine climate region; (**F**) Temperate continental climate region (**G**) Temperate monsoon climate region; (**H**) Subtropical monsoon climate region.

Figure 5 presents the seasonal analysis of the PRCPTOT index. In spring, except for the plateau alpine climate region, the peak value of the PRCPTOT index for other climate regions and the FMB shifts to the right compared to the historical data, and the density change is small. The peak value in the plateau and alpine climate zone in spring was approximately 100 mm. The future period scenarios show a significant decreasing trend in the following order: SSP1-2.6 > SSP3-7.0 > SSP5-8.5. In winters, there is minimal difference in the probability distribution between the historical period versus the future period. In autumn, the probability distribution of each climate region is distinct. The plateau alpine climate region and the temperate continental climate show a distribution like that of spring. The difference between the historical and future periods is minimal in the temperate monsoon climate region. However, in the subtropical monsoon climate region, the peak value shifts about 150 mm to the left. This indicates that PRCPTOT is the index most sensitive to climate change in spring and autumn, and confirms that climate warming can promote the intensification of precipitation processes with the increase of carbon emissions.

**Figure 6:**
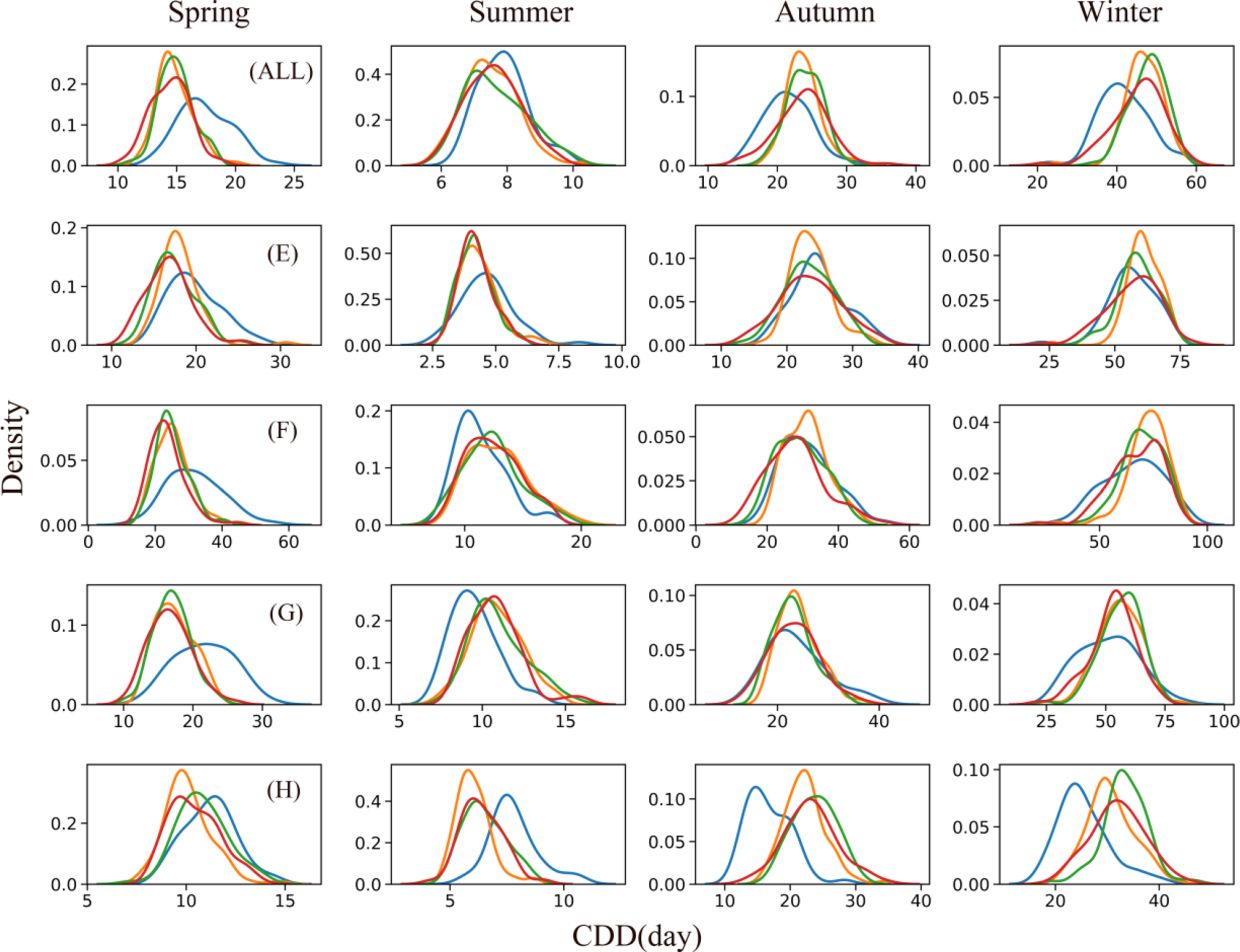
Probability density map of CDD in different seasons (Blue is hist scenario, orange is SSP1-2.6 scenario, green is SSP3-7.0, and red is SSP5-8.5 scenario) (ALL) FMB; (**E**) Plateau alpine climate region; (**F**) Temperate continental climate region (**G**) Temperate monsoon climate region (**H**) Subtropical monsoon climate region.

Figure 6 displays the CDD index from a seasonal perspective. In spring, the peak of CDD shifted to the right by 5 days. It was relatively less affected by climate change in the temperate monsoon and temperate continental climates in the future period. In summer, the peak value of CDD shifted to the left and the density increased in the plateau alpine and subtropical monsoon climates, while that of the temperate continental and temperate monsoon climates showed an opposite trend In autumn, only the tropical monsoon climate had a significant change, with the peak value shifted to the right approximately 13 d. In the winter tropical monsoon climate zone, the peak value shifted to the right, but the density remained constant. In the other climate zones, the peak value was similar, but the density increased. These results suggest that drought risks will decrease in spring, then increase in the temperate climate during summer and increase in the subtropical monsoon climate during autumn and winter, which has important implications for regional water resource management and drought mitigation strategies.

**Figure 7:**
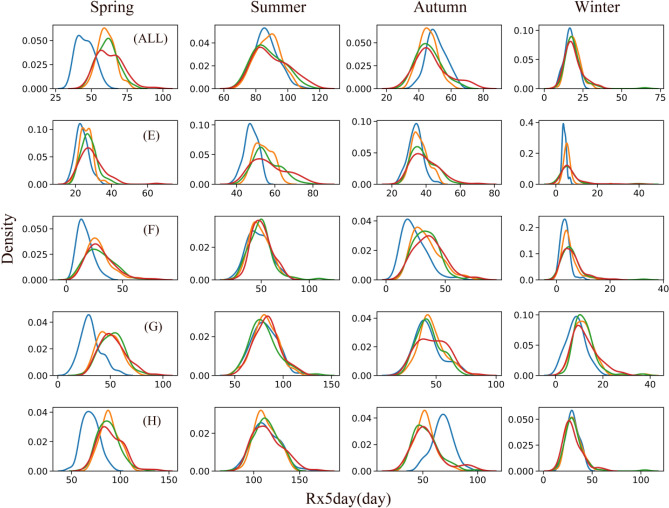
Probability density map of Rx5day in different seasons (Blue is hist scenario, orange is SSP1-2.6 scenario, green is SSP3-7.0, and red is SSP5-8.5 scenario) (ALL)FMB; (**E**) Plateau alpine climate region; (**F**) Temperate continental climate region (**G**) Temperate monsoon climate region; (**H**) Subtropical monsoon climate region.

Figure 7 illustrates the Rx5day index from a seasonal perspective. The pattern of variation for Rx5day was similar to that of PRCPTOT, with the main difference being that the peak probability of Rx5day was substantially higher than that of PRCPTOT, and more concentrated on the peak value. The degree of variation for the Rx5day index was highest in autumn, with a longer tail in winter, and the probability of occurrence in the historical period was substantially higher than that of future period. These findings suggest that, compared to historical periods, the likelihood of flooding in the FMB in the future is considerably higher in spring and summer in the plateau and alpine climate, higher in autumn in the temperate monsoon and temperate continental climates for the SSP5-8.5 scenario, and lower in the subtropical monsoon climate zone.

### Future correlation of extreme precipitation

**Figure 8:**
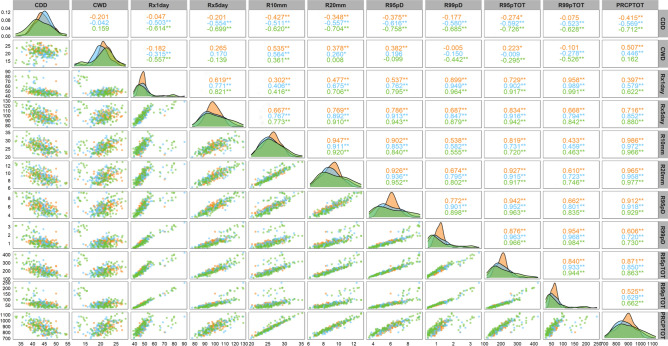
Correlation between extreme precipitation indexes: the lower part is the kernel density estimation, the diagonal is the histogram, and the upper part is the Pearson correlation coefficient, * means pass the 95% significance test, ** means pass the 99% significance test.

The intensity, frequency, and duration of precipitation are crucial that impact the total precipitation change. In this study, we analyse the correlation among the indices in the future and conducted further analysis on the extreme precipitation indices leading to precipitation in the FMB from 2015 to 2100. As shown in Fig. 8, each index will show correlations different from PRCPTOT. Notably, CDD will display a significant negative correlation with the PRCPTOT index. In particular, the SSP1-2.6 and SSP3-7.0 of CWD will show no statistical significance with PRCPTOT at the confidence level of 0.05, while other indices will show significant positive correlations.

CDD will be negatively correlated with PRCPTOT in different scenarios. Different scenarios of correlation between Rx1day and PRCPTOT will be weak correlation, moderate correlation, and strong correlation, respectively. Rx5day, R10mm, R20mm, R95pTOT, R95pD, R99pTOT, and R99pD will show strong correlation with PRCPTOT, with the correlation value order of: SSP1-2.6 < SSP3-7.0 < SSP5-8.5. The findings indicate that, under the background of climate change, an increase in precipitation will be accompanied by an increase in the extreme precipitation index and a decrease in the CDD index. The results show that the increase of R10mm corresponds to an increase of annual precipitation.

### Influence of regional geographic factors on extreme precipitation index

**Figure 9:**
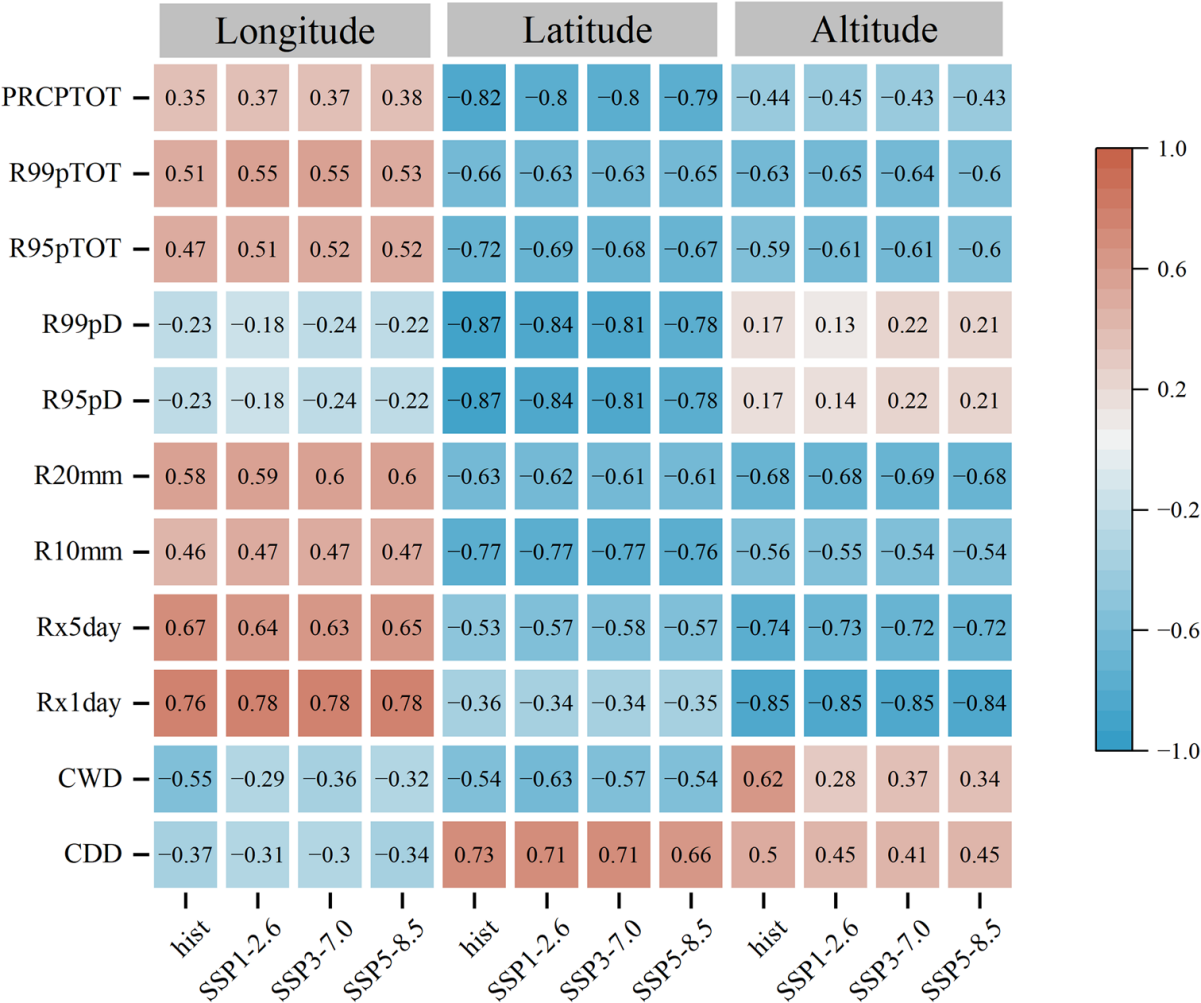
Correlation coefficient between extreme precipitation index and longitude, latitude, and altitude.

The potential influence of geographical factors such as longitude, latitude, and altitude on the extreme precipitation index is further analysed using the Pearson correlation coefficient. As shown in Fig. 9, both historical and future extreme indices are significantly correlated with longitude, latitude, and altitude (*P* < 0.01). CDD, CWD, R95pD, and R99pD are negatively correlated with longitude, and the correlation in the future is lower than that in the historical. On the other hand, other indices show a positive correlation with longitude. The correlation coefficients of Rx1day and Rx5day are found to be high, with maximum values of 0.78 and 0.67, respectively. In the examined scenarios, the correlation coefficients increase in the order: SSP1-2.6 < SSP3-7.0 < SSP5-8.5, which suggests that climate warming is sensitive to geographical factors.

As latitude increases, the amount of solar radiation received by the Earth decreases, which can affect precipitation patterns. CDD have a significant positive correlation with latitude, while the correlation coefficient will decrease with an increase in carbon emissions in the future. R95pD, R99pD, and PRCPTOT have a high positive correlation with latitude, with the maximum correlation coefficients were 0.87, 0.87, and 0.82, respectively. The correlation coefficients of other indices, except for Rx5day and R99pTOT, show a downward trend with an increase in carbon emissions. These results suggest that latitude plays an important role in determining extreme precipitation patterns in the FMB.

Elevation is an important topographic factor that influences precipitation by affecting water vapor and heat. The study area encompasses plains, hills, mountains, and other terrain, and is categorized into elevation zones: < 500 m, 500–1000 m, 1000–1500 m, 1500–3000 m, and > 3000 m. Ach zone exhibits unique extreme precipitation indices, CDD, CWD, R95pD, and R99pD showing positive correlation, while other indices showing negative correlation. Rx1day have the highest correlation, with a maximum correlation coefficient of − 0.85. The indices demonstrating negative correlation remain consistent across historical and future periods, while those showing positive correlation demonstrated a decreasing trend. At elevations less than 500 m, except for PRCPTOT during the historical period (the correlation was tested by 95% significance test), only CDD and Rx1day show negative correlation, while the other indices showed positive correlation. The correlation coefficients of CWD, R95pD, and R99pD were high. In the 500–1000 m and 1000–1500 m elevation zones, all indices except for CDD pass the 95% significance test, with negative correlation coefficients. The correlation coefficient for the 500–1000 m elevation zone is higher than that for the 1000–1500 m zone. At 1500–3000 m, the indices for R95pTOT, R99pTOT, and CDD, during the historical and for Rx5day during future period did not pass the 95% significance test. CDD, Rx1day, Rx5day, R20mm, and R99pTOT are negatively correlated, and the other indices were positively correlated. The correlation coefficients of CWD, R95pD, and R99pD are large. When the altitude was greater than 3000 m, only the CWD index during the historical period failed to pass the 95% significance test. CDD and CWD period are positively correlated, and the correlation coefficient, with the highest correlation coefficient. Overall, the results show that the correlation between extreme precipitation index and altitude is most pronounced at altitudes greater than 3000 m, followed by altitudes less than 500 m. The correlation coefficients for other altitude increase according to the order: 500–1000 m < 1000–1500 m < 1500–3000 m. It is notable that the relationship between extreme precipitation index and altitude differs across the elevation zones. The results highlight the significant effect of altitude on extreme precipitation changes in the FMB.

## Discussion

### Changes of extreme precipitation in different regions

Global warming exacerbates the hydrological cycle, resulting in more frequent and intense extreme precipitation events. However, studies investigating extreme precipitation in the FMB region are scarce. Previous studies focused on individual basins within the FMB, making this study unique in its approach to examining extreme climate events and climate change across the entire region.

At the scale of basin, PRCPTOT, R20mm, Rx5day, R95pD, and R95pTOT showed a gradual increase from northwest to southeast, while CDD displayed the opposite trend. Additionally, CWD in the YTRB was found to be larger than that in the Huang–Huai–Hai Basin. These findings are consistent with the work of Wang^29^ despite differences in the data used.

Extreme precipitation changes across different climate zones exhibit varying degrees of spatial heterogeneity. This study found that, under the context of climate change, extreme precipitation in the plateau and alpine climate zone showed an increasing trend except for CDD, which was found to be more sensitive to climate change than other indices. It shows that the duration of extreme precipitation increases more significantly in this region under the background of global warming. The extreme precipitation index of temperate continental climate zone and temperate monsoon climate zone remained relatively stable with the former having lower values than the latter. Meanwhile, the extreme precipitation index of subtropical monsoon climatic zone has the largest change, and extreme precipitation events are more frequent, while the changes of CDD and CWD are more significant. It is worth noting that Rx5day shows a decreasing trend under the influence of climate change in this region. These trend in extreme precipitation events have significant implications for water resources management in the future, particularly in the subtropical monsoon climate region.

### Causes of temporal variation in extreme precipitation

**Figure 10:**
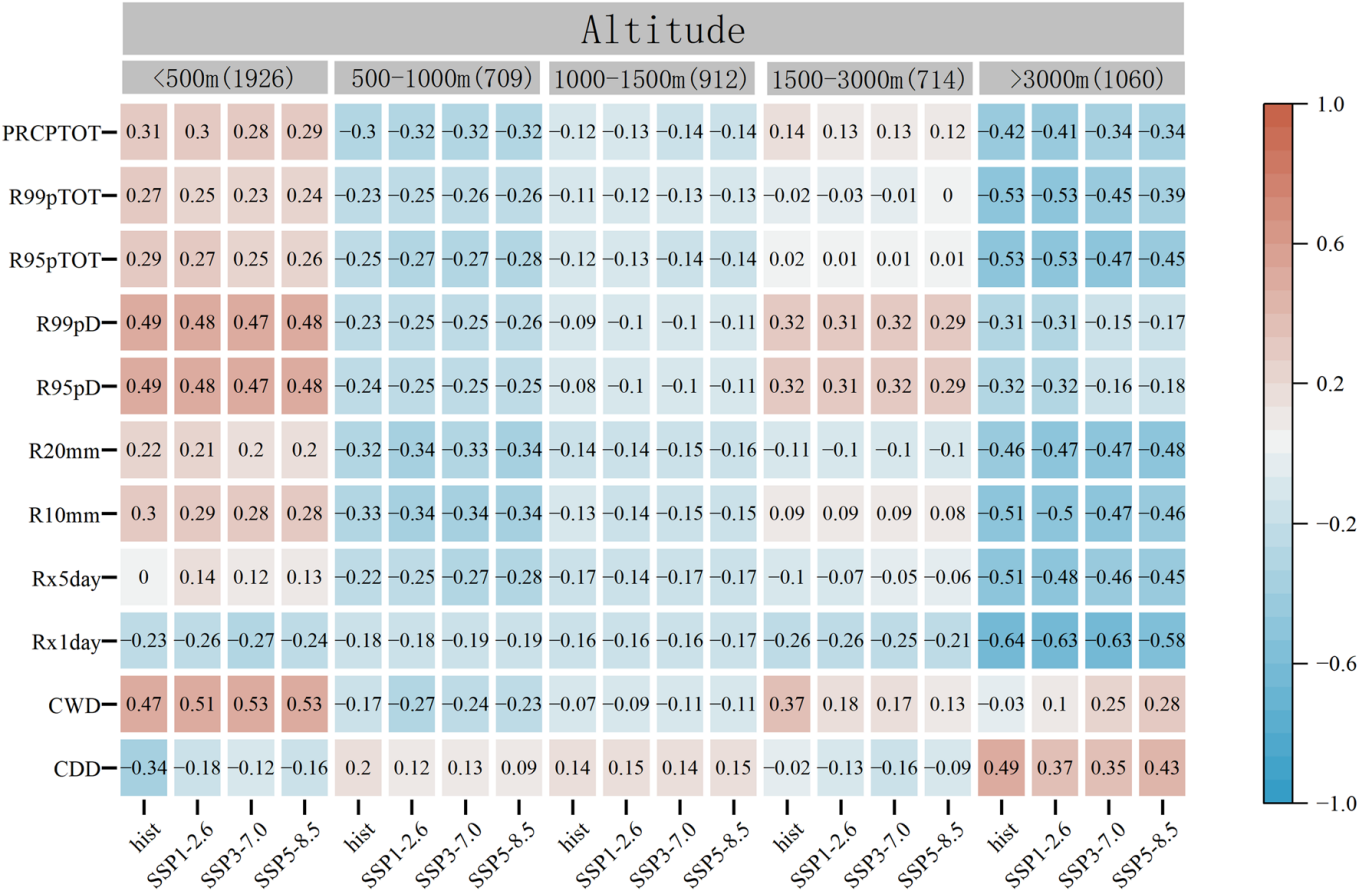
Correlation between different altitudes and extreme precipitation index.

This study examines the regional characteristics of extreme precipitation by analysing historical observation data and data from three different SSP scenarios (SSP1-2.6, SSP3-7.0, and SSP5-8.5) for 11 extreme precipitation indices. First, the temporal and spatial variation trend of past and future extreme precipitation indices were analysed. Then, the seasonal variation of future extreme precipitation indices and their correlations. As shown in Fig. 10, from 1960 to 2014, CDD and R99pTOT exhibited an increasing trend, while PRCPTOT showed a decreasing trend. Other indices show only minor changes. However, under the influence of climate change, all extreme precipitation indices are expected to change significantly in the future, except for CDD, which is expected to show an increasing trend. Furthermore, the study found that the precipitation risk of floods is expected to increase substantially in spring and in the plateau and alpine climate zones, while it is expected to decrease in summer in the temperate zone. The risk is also expected to increase in autumn and winter in the subtropical monsoon climate zone, although the flood risk is predicted to decrease.

### Correlation with atmospheric circulation factors

Extreme precipitation is influenced by regional atmospheric circulation pattern and the general characteristics of climate system. The change of extreme precipitation in FMB is very complex due to the region’s multiple climatic zones, which are affected differently by atmospheric circulation. To better understand these patterns, calculated the correlation between extreme precipitation indices and ENSO, Nino 3.4, PDO, AO, AMO, NAO, SOI and EASMI, SASMI and SCSSMI.

**Figure 11:**
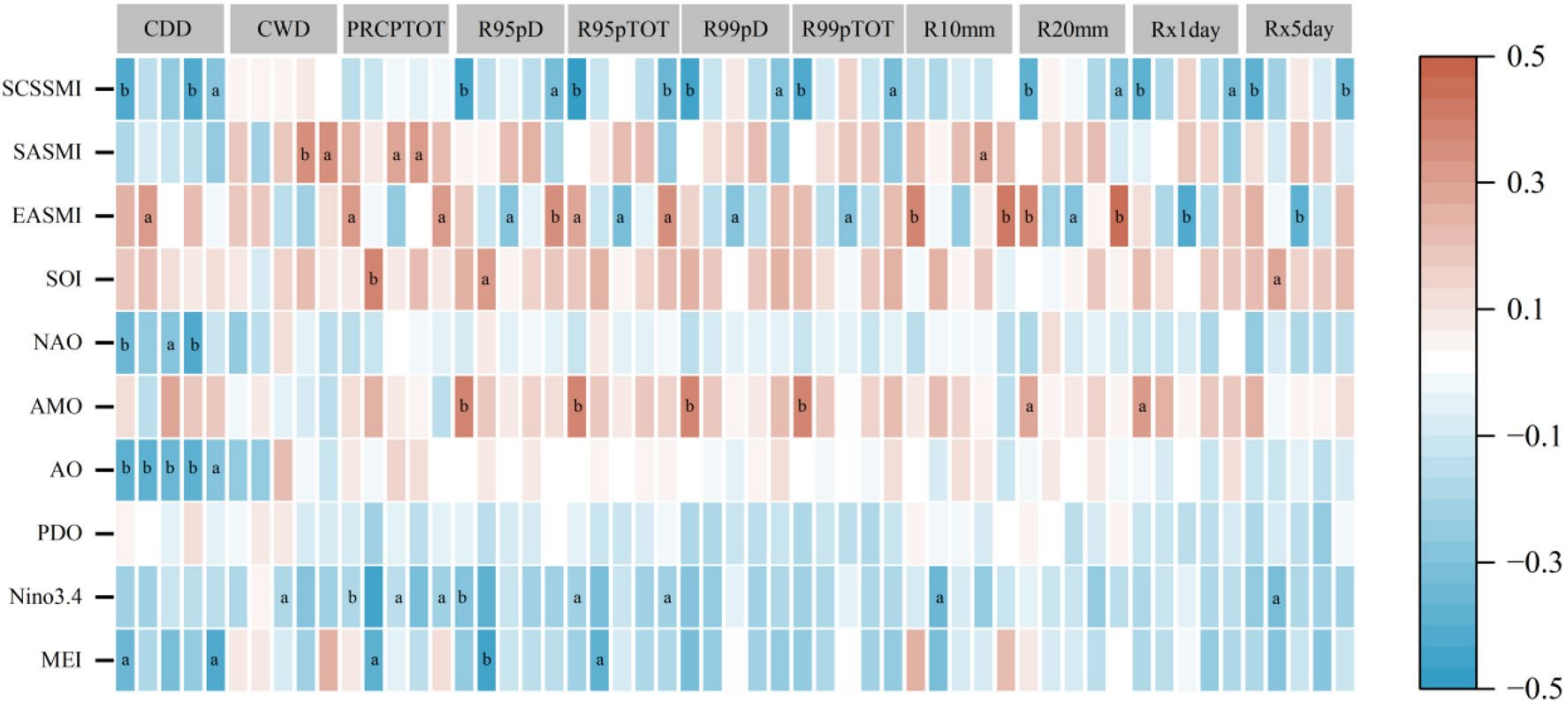
Correlation between atmospheric circulation patterns and extreme precipitation index over the period of 1961–2014. *Note* **a** is the 0.05 confidence level, and **b** is the 0.01 confidence level. The five columns of correlations under each index are FMB, E, F, G, and H regions, respectively.

ENSO can cause anomalies in atmospheric circulation patterns, leading to significant impacts on regional extreme precipitation worldwide, including China^42–44^. As shown in Fig. 11, our study shows that Nino 3.4 is significantly correlated with extreme precipitation events in FMB (PRCPTOT, R95pD, R95pTOT), Temperate continental climate region (CWD, PRCPTOT), Plateau alpine climate region (R10mm, Rx5day) and Subtropical monsoon climate region (PRCPTOT, R95pTOT). MEI is significantly correlated with CDD in subtropical monsoon climate and PRCPTOT, R95pD and R95pTOT in plateau alpine climate. EASMI, SASMI and SCSSMI were more significantly correlated with extreme precipitation events in FMB. EASMI was mainly reflected in temperate continental climate zone and subtropical monsoon climate zone. SASMI is mainly found in temperate monsoon climates (CWD, PRCPTOT, R10). Except PRCPTOT and CWD, SCSSMI was significantly correlated with other extreme precipitation indices of FMB, especially in the subtropical monsoon climate region. There was no significant correlation between PDO and the extreme precipitation index of FMB, there was a significant correlation between AO and CDD in each FMB climate zone. AMO was significantly correlated with R95pD, R99pD, R95pTOT, R99pTOT, R20mm and Rx1day of FMB. NAO was significantly correlated with CDD in temperate and subtropical monsoon climates. SOI was significantly correlated with PRCPTOT, R95pD and Rx5day. In summary, FMB is affected by a various atmospheric circulation factors, with different correlations in different climate zones. These findings provide valuable insights for predicting the future trend of extreme precipitation events.

### Correlation with geographical factors

Geographical factors play a significant role in the variation of extreme precipitation our study shows that longitude and latitude of the FMB are highly correlated with the variation trend of extreme precipitation. This could be attributed to the amplification of thermal characteristics between land and ocean under the background of climate warming. Latitude was found to have a strong influence on CDD, CWD, R95pD, R99pD, and PRCPTOT of the FMB. While longitude had a significant impact on Rx1day and Rx5day. The joint effect of latitude and longitude was observed for R10mm, R20mm, R95pTOT, and R99pTOT. Topography is another factor affecting the spatial distribution of extreme precipitation. Different terrains have different responses to extreme precipitation. In areas with altitudes less than 500 m, extreme precipitation indices were positively correlated except for CDD and CWD. In areas with altitudes of 500–1500 m, extreme precipitation indices were negatively correlated except for CDD. In areas with altitudes more than 3000 m, extreme precipitation indices were negatively correlated except for CDD. CDD, Rx1day, R10mm, R95pTOT, and R99pTOT were highly correlated.

## Conclusions

Our research suggests that climate change is significantly affecting the hydrological cycle and leading to changes in of extreme weather patterns. In this study, extreme precipitation indices (CDD, CWD, Rx1day, Rx5day, R10mm, R20mm, R95pD, R95pTOT, R99pTOT, and PRCPTOT) were used to study the changes of extreme precipitation in the FMB based on observational data and CMIP6 model simulation. The main conclusions can be summarized as follows:

1. For the mean temporal trend of the FMB, all extreme indices except CDD showed an upward trend during 1960–2100, indicating that that climate warming has intensified the precipitation process. This increase in extreme precipitation is expected to become more significant with further temperature rise. Furthermore, although CDD decreased, but annual total precipitation increased, indicating that the increase of precipitation should be related to the increase of precipitation days. The study also projected changes in extreme precipitation indices under different climate scenarios by 2100. Compared to SSP1-2.6, the change rates of SSP5-8.5 are projected to be: CDD (84%) and R99pTOT (205%), while the change rates of other indices are projected to be approximately 110%. Compared with SSP1-2.6, the change rates of SSP3-7.0 are CDD (96%) and R99pTOT (130%). The change rate of other indices is approximately 103%.
2. In terms of spatial trends, PRCPTOT, R20mm, Rx5day, R95pD, and R95pTOT showed a gradual increase from northwest to southeast in the FMB, while CDD displayed the opposite trend. Climate change had a significant impact on extreme precipitation in the plateau alpine and subtropical monsoon climate regions. Moreover, the sensitivity of extreme precipitation indices to climate change varied among seasons and climate zones. In spring and autumn, PRCPTOT, CDD, and Rx5day were the most sensitive to climate change. In temperate continental and temperate monsoon climate zones, drought risk decreased in spring, but increased in autumn and winter, while drought risk increased in summer.
3. The correlations between extreme precipitation indices in the FMB indicates that each index is significantly correlated with PRCPTOT in the future period. The increase in precipitation will increase the probability of extreme precipitation events. The influence on CDD and Rx1day was particularly significant. The correlation between extreme precipitation index and atmospheric circulation shows that ENSO and monsoon climate significantly affect extreme precipitation index in different climate regions of FMB.
4. Correlation analysis between the extreme precipitation index and geographical location suggests that latitude has a significant impact on CDD, CWD, R95pD, R99pD, and PRCPTOT; longitude has a significant influence on Rx1day and Rx5day; and both latitude and longitude jointly impact R10mm, R20mm, R95pTOT, and R99pTOT. The effect of altitude on the extreme precipitation index is complex, with the greatest impact observed above 3000 m. In contrast, minimal influence was noted on the extreme precipitation index between 500 and 3000 m, indicating that high altitude areas are more sensitive to climate change.

## Data Availability

The daily precipitation data from meteorological stations were obtained from the National Meteorological Science Data Centre (https://data.cma.cn/). The CMIP6 output is available from the Earth System Grid Federation (https://esgf-node.llnl.gov/projects/cmip6/). The large-scale atmospheric circulation patterns from the Earth System Research Laboratory of the Physical Sciences Division of the United States National Oceanic and atmospheric administration (https://www.esrl.noaa.gov/psd/data/climateindices/list/). The East Asian summer monsoon Index (EASMI) and South China Sea summer monsoon index (SCSSMI) from http://lijianping.cn/dct/page/1. More analysed during the current study available from the corresponding author on reasonable request.

## Code availability

Custom code generated in this study is available at https://github.com/usman-steven/extreme-precipitation.

## References

[1] Gao T, Xie L (2016). Spatiotemporal changes in precipitation extremes over Yangtze River basin, China, considering the rainfall shift in the late 1970s. Glob.Planet. Change.

[2] Cui H (2022). Dynamics and potential synchronization of regional precipitation concentration and drought-flood abrupt alternation under the influence of reservoir climate. J. Hydrol. Reg. Stud..

[3] Hady AA (2013). Deep solar minimum and global climate changes. J. Adv. Res..

[4] Hui Z (2018). Global warming and rainfall: Lessons from an analysis of Mid-Miocene climate data. Palaeogeogr Palaeocl.

[5] Liu YR, Li YP, Yang X, Huang GH, Li YF (2021). Development of an integrated multivariate trend-frequency analysis method: Spatial-temporal characteristics of climate extremes under global warming for Central Asia. Environ. Res.

[6] Pfahl S, O’Gorman PA, Fischer EM (2017). Understanding the regional pattern of projected future changes in extreme precipitation. Nat. Clim. Change.

[7] Gentilucci M, Barbieri M, D’Aprile F, Zardi D (2020). Analysis of extreme precipitation indices in the Marche region (central Italy), combined with the assessment of energy implications and hydrogeological risk. Energy Rep..

[8] Gershunov A, Benmarhnia T, Aguilera R (2018). Human health implications of extreme precipitation events and water quality in California, USA: A canonical correlation analysis. Lancet Planet. Health.

[9] Knapp AK (2008). Consequences of more extreme precipitation regimes for terrestrial ecosystems. Bioscience.

[10] Lu M (2019). Effect of urbanisation on extreme precipitation based on nonstationary models in the Yangtze River Delta metropolitan region. Sci. Total Environ..

[11] Zhi (2011). Assessing the site-specific impacts of climate change on hydrology, soil erosion and crop yields in the Loess Plateau of China. Clim. Change.

[12] Powell JP, Reinhard S (2016). Measuring the effects of extreme weather events on yields. Weather Clim. Extremes.

[13] Cavalcanti IFA (2015). Precipitation extremes over La Plata Basin: Review and new results from observations and climate simulations. J. Hydrol..

[14] Subash N, Singh SS, Priya N (2011). Extreme rainfall indices and its impact on rice productivity: A case study over sub-humid climatic environment. Agric. Water Manag..

[15] Pachauri K, Meyer A (2014). Climate change 2014. Synth. Rep..

[16] Moccia B, Papalexiou SM, Russo F, Napolitano F (2021). Spatial variability of precipitation extremes over Italy using a fine-resolution gridded product. J. Hydrol. Reg. Stud..

[17] Olmo M, Bettolli ML, Rusticucci M (2020). Atmospheric circulation influence on temperature and precipitation individual and compound daily extreme events: Spatial variability and trends over southern South America. Weather Clim. Extremes.

[18] Talchabhadel R, Karki R, Thapa BR, Maharjan M, Parajuli B (2018). Spatio-temporal variability of extreme precipitation in Nepal. Int. J. Climatol..

[19] Tong S (2018). Spatial and temporal variability in extreme temperature and precipitation events in Inner Mongolia (China) during 1960–2017. Sci. Total Environ..

[20] Pei F (2018). Detection and attribution of extreme precipitation changes from 1961 to 2012 in the Yangtze River Delta in China. CATENA.

[21] Gao L, Huang J, Chen X, Chen Y, Liu M (2018). Contributions of natural climate changes and human activities to the trend of extreme precipitation. Atmos. Res..

[22] Zheng W, Wang S (2021). Extreme precipitation accelerates the contribution of nitrate sources from anthropogenetic activities to groundwater in a typical headwater area of the North China Plain. J. Hydrol..

[23] Tabari H (2020). Climate change impact on flood and extreme precipitation increases with water availability. Sci. Rep..

[24] Mingzhong (2017). Spatiotemporal variations of extreme precipitation regimes during 1961–2010 and possible teleconnections with climate indices across China. Int. J. Climatol..

[25] Li W, Xiaogang HE, Scaioni M, Yao D, Li X (2019). Annual precipitation and daily extreme precipitation distribution: Possible trends from 1960 to 2010 in urban areas of China. Geom. Nat. Hazards Risk.

[26] Xie Y, Xing J, Shi J, Dou Y, Lei Y (2016). Impacts of radiance data assimilation on the Beijing 721 heavy rainfall. Atmos. Res..

[27] Hsu P-C (2023). Multiscale interactions driving the devastating floods in Henan Province, China during July 2021. Weather Clim. Extremes.

[28] Ning L, Qian Y (2009). Interdecadal change in extreme precipitation over South China and its mechanism. Adv. Atmos. Sci.

[29] Wang G, Zhang Q, Yu H, Shen Z, Sun P (2020). Double increase in precipitation extremes across China in a 1.5 °C/2.0 °C warmer climate. Sci. Total Environ..

[30] Yu M, Wang C, Liu Y, Olsson G, Wang C (2018). Sustainability of mega water diversion projects: Experience and lessons from China. Sci. Total Environ..

[31] Wang B (2013). Changes in extreme precipitation over Northeast China, 1960–2011. Quat. Int..

[32] Zhang C (2020). Moisture sources for precipitation in Southwest China in summer and the changes during the extreme droughts of 2006 and 2011. J. Hydrol..

[33] Tebaldi C, Debeire K, Eyring V, Fischer EM, Ziehn T (2021). Climate model projections from the Scenario Model Intercomparison Project (ScenarioMIP) of CMIP6. Earth Syst. Dyn..

[34] Guo B, Zhang J, Meng X, Xu T, Song Y (2020). Long-term spatio-temporal precipitation variations in China with precipitation surface interpolated by ANUSPLIN. Sci. Rep..

[35] Wang, S., Wang, D. & Huang, C. A comparative study of using ANUSPLIN and GWR models for downscaled GPM precipitation. In *2019 8th International Conference on Agro-Geoinformatics.* DOI: 10.1109/Agro-Geoinformatics (2019).

[36] Penny WD, Mattout J, Trujillo-Barreto N (2007). CHAPTER 35: Bayesian model selection and averaging. Stat. Parametr. Mapp..

[37] Miao C, Su L, Sun Q, Duan Q (2016). A nonstationary bias-correction technique to remove bias in GCM simulations: Bias-correction in the GCM simulation. J. Geophys. Res-Atmos.

[38] Sen PK (1968). Estimates of the regression coefficient based on Kendall's Tau. J. Am. Stat. Assoc..

[39] Yue S, Wang CY (2004). The Mann-Kendall test modified by effective sample size to detect trend in serially correlated hydrological series. Water. Resour. Manag..

[40] Sang Y-F, Wang Z, Liu C (2014). Comparison of the MK test and EMD method for trend identification in hydrological time series. J. Hydrol..

[41] Naikoo MW, Talukdar S, Das T, Rahman A (2022). Identification of homogenous rainfall regions with trend analysis using fuzzy logic and clustering approach coupled with advanced trend analysis techniques in Mumbai city. Urban Clim..

[42] Wang D (2022). Spatiotemporal variability of extreme precipitation at different time scales and quantitative analysis of associated driving teleconnection factors: Insights from Taihu Basin, China. Ecol. Indic..

[43] Li P, Yu Z, Jiang P, Wu C (2021). Spatiotemporal characteristics of regional extreme precipitation in Yangtze River Basin. J. Hydrol..

[44] Song X (2019). Changes in precipitation extremes in the Beijing metropolitan area during 1960–2012. Atmos. Res..

